# Material Deprivation, Psychological Distress, and Substance Use Among Socioeconomically Disadvantaged Adolescents: The Mediating Roles of Social Relationships

**DOI:** 10.1002/bsl.70058

**Published:** 2026-03-24

**Authors:** Zewei Liu, Ji‐Kang Chen

**Affiliations:** ^1^ The Chinese University of Hong Kong Sha Tin Hong Kong

**Keywords:** behavioral health, interpersonal relationships, juvenile delinquency, mental health, poverty, socioeconomic determinants

## Abstract

Previous research on the mediating role of the quality of social relationships between material deprivation and adolescent health has predominantly utilized samples from mainstream populations to examine mental health outcomes, neglecting the critical need to explore behavioral health outcomes, such as substance use, particularly in socioeconomically disadvantaged adolescents. Using structural equation modeling with data from 1329 Taiwanese adolescents living in poverty and receiving social assistance, the study found that the quality of social relationships with family members, teachers, and peers significantly mediated the association between material deprivation and substance use. The family–adolescent relationship quality significantly mediated the association between material deprivation and psychological distress, while the quality of peer and teacher–student relationships did not. The mediating mechanisms of social relationships are similar across different age subgroups and sexes. This study informs practices addressing psychological distress and substance use issues via family and relational pathways.

## 1 Introduction

Adolescence is a crucial developmental period during which externalizing and internalizing problems, such as misconduct and distress, can adversely affect overall health and well‐being. Adolescent substance use, such as alcohol and drug use, is an externalizing problematic behavior linked with other delinquent and health‐related problems, warranting extensive research (Yu and Chen 2025). Adolescent psychological distress, reflecting internalizing suffering and unhealthy emotional conditions, is another critical issue that exacerbates developmental problems (Tian et al. 2021). Substance use and psychological distress during adolescence can negatively impact performance in both academic and life domains, which is also linked with family dysfunction, increased involvement with justice systems, and an elevated risk of developing mental disorders later in life (Simon et al. 2022). Given that these concerns exist globally, researchers have called for a thorough investigation of determinants and pathways to inform promotion, prevention, and intervention efforts for protecting adolescents from harm (Tian et al. 2021).

Material deprivation, the lack of essential material resources evaluated by both socioeconomic conditions and personal needs, is considered an important factor in increasing adolescents' risks of engaging in substance use and experiencing psychological distress (Conger et al. 2010; Gross‐Manos and Ben‐Arieh 2017). Adolescents may be deprived of necessities for basic life and development, such as food, water, clothing, electricity, places to study, medical costs, and pocket money, which reflects the material pressure they face and possibly leads to externalizing and internalizing problems. As such, researchers have explored psychosocial pathways from material deprivation to its outcomes (J. K. Chen, Wang, et al. 2021; Yoshikawa et al. 2012). Following this, the quality of social interactions with family members, teachers, and peers has been considered the underlying mediating pathways (J. K. Chen et al. 2023; Lau and Bradshaw 2016). However, previous studies have focused on psychological outcomes using samples from mainstream or socially privileged adolescent populations. This line of literature overlooks whether the quality of social interactions can explain the links between material deprivation and adolescents' substance use, and whether the mediating pathways apply to socioeconomically disadvantaged adolescents.

Some adolescents resort to substance use or experience psychological distress due to avoidant coping with material and interpersonal stress (Baumann et al. 2007; J. K. Chen, Wang, et al. 2021; Knyazev 2004; Lee et al. 2017). Researchers have found that low‐quality social relationships are linked with substance use and psychological distress among adolescents (Hong et al. 2010, 2011; J. K. Chen et al. 2023). This issue is particularly relevant for socioeconomically disadvantaged adolescents, as they are more likely to experience the negative effects of material deprivation and strained social relationships, and thus may show more substance use and psychological distress (Dong et al. 2024; Hong et al. 2024; Makarios et al. 2017; Martin et al. 2015; Reiss 2013). However, it remains unclear whether social relationship quality can explain the interrelationships between material deprivation and both psychological distress and substance use among socioeconomically disadvantaged adolescents. Using survey data from Taiwanese adolescents living in poverty and receiving social assistance, this study thus aims to examine the mediating roles of the quality of family–adolescent relationships, peer relationships, and teacher–student relationships in the links of material deprivation with substance use and psychological distress. It also explores whether the interrelationships among these variables are sustained across age and sex subgroups.

## 2 Literature Review

### 2.1 Material Deprivation, Psychological Distress, and Substance Use

Material deprivation has been regarded as a determinant of adolescent mental and behavioral health, providing evaluations about both socioeconomic conditions and personal needs from the adolescent view, yet whether the associations are significant or not remains mixed in empirical studies (J. K. Chen, Wang, et al. 2021). Some researchers have found that adolescents deprived of material resources are more likely to engage in substance use as a means of coping with their stressors (Baumann et al. 2007; Coley et al. 2017). However, other studies show that material deprivation does not necessarily lead to psychological distress and substance use (J. K. Chen et al. 2023; Cho 2018); for example, adolescents from families with worse socioeconomic conditions may have insufficient material resources for additional expenses, limiting their access to alcohol, cigarettes, and drugs, reducing the likelihood of engaging in substance use (Hai et al. 2024; Piko and Fitzpatrick 2007; Ritterman et al. 2009). Researchers have further called for a need to explicitly explore the underlying mechanisms that link material deprivation to adolescent psychological distress and substance use, given these abovementioned mixed findings (Khin et al. 2024; Lee et al. 2017).

### 2.2 Social Relationships as Potential Mediating Mechanisms

Psychosocial factors, particularly the quality of social relationships, are crucial for understanding pathways to holistic health and well‐being outcomes across different socioeconomic backgrounds (Adler and Stewart 2010; Braveman et al. 2011). Researchers have regarded social relationship quality as a potential mediator between material deprivation and adolescents' mental and behavioral health (Yoshikawa et al. 2012). For example, materially deprived adolescents tend to have lower levels of social competence and emotional regulation (Gross‐Manos and Ben‐Arieh 2017; Hjalmarsson and Mood 2015); and as their difficulties in interpersonal interactions increase, they are more likely to experience peer rejection or social isolation, thus exacerbating their cognitive‐behavioral avoidance (Knyazev 2004; J. K. Chen, Wang, et al. 2021). In particular, given that adolescents' social interactions are strongly embedded within family and school systems, the quality of relationships with family members, peers, and teachers plays a significant role in shaping their health and well‐being (J. K. Chen et al. 2021, 2023, 2025; He et al. 2025; Steinberg 2001). A few empirical studies have tested the above observations and mechanisms, demonstrating that social relationship quality mediates the association between material deprivation and adolescent health (J. K. Chen et al. 2023; Lau and Bradshaw 2016). Yet these studies have paid attention to psychological outcomes instead of behavioral problems, such as substance use.

Based on theory and evidence, we argue that the mediating role of the quality of social relationships in the link between material deprivation and adolescent outcomes can be applied to substance use. First, according to theoretical discussions on social relationships, substance use can be regarded as behavioral compensation and avoidance for coping with strain in social interactions and stress from a lack of material resources (Baumann et al. 2007; J. K. Chen, Wang, et al. 2021; Lee et al. 2017). Second, empirical studies have reported significant direct effects of social and material deprivation on tobacco, alcohol, and psychotropic drug use (Baumann et al. 2007; Espelage et al. 2013). Third, higher levels of material deprivation have been found to be significantly linked with poorer social relationship quality (J. K. Chen et al. 2023; Gross‐Manos and Ben‐Arieh 2017). Fourth, an ecological review of the literature has supported that interactions with family members, teachers, and peers are important microsystems in shaping adolescents' alcohol and tobacco use (Hong et al. 2011). However, limited studies have thoroughly examined the pathways mentioned above within a comprehensive model. Examining these pathways could help clarify how the quality of social relationships is associated with material deprivation and substance use. This research may help us extend our understanding of mediating roles of social relationships in relation to behavioral outcomes, an area of research that has rarely been emphasized in previous studies. By doing so, we can provide empirical evidence to support more complex modeling and inform behavioral health‐related practice for adolescents experiencing material deprivation.

Furthermore, previous studies have focused on mainstream or socially privileged adolescents (J. K. Chen et al. 2023; Lau and Bradshaw 2016), highlighting the need for further investigations among vulnerable groups, particularly socioeconomically disadvantaged adolescents. Such vulnerable groups experience intersectional and cumulative disadvantages that can amplify the negative impact of material deprivation and complicate the mediating mechanisms of social relationships (Coley et al. 2017; Yuan et al. 2022). In addition, adolescents may fall into socioeconomically disadvantaged situations for various reasons, such as parental unemployment, low parental income, loss of family members, non‐intact family care, migration, and homelessness (Ratcliff et al. 2025; Yuan et al. 2022). Although the social welfare system (in universal or selective forms) aids some people in need, welfare conditionality, resource accessibility, and systemic barriers may still prevent marginalized adolescents from leading a quality life as their counterparts do (Gennetian et al. 2004; Hong et al. 2024; Vidal et al. 2016). Moreover, socioeconomically disadvantaged adolescents are more susceptible to adverse material and financial conditions, increasing their risk of substance use and psychological distress due to a lack of supportive resources and poor social competencies (Dong et al. 2024; Hong et al. 2010, 2024; Martin et al. 2015). However, there remains a lack of studies examining the mediating mechanisms of social relationship quality in the associations of material deprivation with adolescent outcomes among socioeconomically disadvantaged groups. This limits theoretical discussions and practical applications for specific minority populations.

In addition, researchers have debated the differences in the above‐described mediating pathways across sex and age subgroups. Such differences have not only been found in the average levels of psychological distress and substance use (Dong et al. 2024; Makarios et al. 2017; O’Malley et al. 2006; Tian et al. 2021), but also in the associations of material deprivation or socioeconomic stressors with adolescents' substance use and psychological well‐being (Gross‐Manos and Ben‐Arieh 2017; Khin et al. 2024; Shah and Watson 2020). On the contrary, another line of literature has documented that the psychosocial pathways in the associations of socioeconomic determinants with adolescent substance use and psychological distress had nonsignificant differences across sex and age subgroups (J. K. Chen et al. 2023; Lee et al. 2017; Reiss 2013). These findings suggest that it is still subject to debate whether the mediating roles of the social relationship quality in the interrelationships between material deprivation and adolescent outcomes among socioeconomically disadvantaged adolescents are sustained across age and sex subgroups. To respond to the research debates, it is necessary to further explore such subgroup heterogeneity and the applicability of the proposed mediating mechanisms, thus informing population‐specific practices.

### 2.3 The Present Study

Using data from socioeconomically disadvantaged adolescents, this study examined the quality of three types of social relationships (family–adolescent relationships, peer relationships, and teacher–student relationships) as mediators in the links of material deprivation with psychological distress and substance use. The study further compared differences in the mediating pathways across age and sex subgroups. We hypothesize that materially deprived adolescents may feature low quality of social relationships, thereby showing higher risks of substance use and psychological distress. This mediation model is assumed to apply to younger and older or female and male adolescents. The study aims to make multifaceted research contributions. Specifically, this study may support the notion that material deprivation is a socioeconomic determinant of adolescent mental and behavioral health by examining its direct and indirect associations via social relationship quality. By doing so, it may enrich the discussion on how the quality of social relationships can act as a psychosocial pathway. Additionally, it seeks to preliminarily extend the roles of social relationships in mediating substance use and contributing to behavioral health promotion. This study may further contribute to population‐specific insights by investigating a vulnerable sample of socioeconomically disadvantaged adolescents.

## 3 Methods

### 3.1 Data and Participants

This study used data from the Taiwan Database of Children and Youth in Poverty (TDCYP), which holds information on a national sample of students from poor families collected using systematic sampling (Taiwan Fund for Children and Families 2014). Informed consent was obtained from parents and their adolescents whose data were included in the database. Over two‐thirds of the participants received governmental assistance for low‐ and lower‐middle‐income households, while others were from poor families recognized by TFCF, a well‐known nonprofit welfare organization in Taiwan. The surveys were completed by clients and social workers at branch offices of the TFCF. We analyzed data from the survey that was conducted in 2013–2014, as it investigated questions relevant to our key variables. The response rate was 71.1%. Respondents completed a self‐administered questionnaire investigating their family's socioeconomic conditions, personal characteristics, and psychological and behavioral outcomes. This study included 1329 adolescents at middle (*n* = 552) and high (*n* = 777) schools who provided complete information on all variables. The mean age of respondents was 15 years, ranging from 10 to 17 years. Half of the respondents were female adolescents (50.34%).

### 3.2 Measures

#### 3.2.1 Material Deprivation

This study employed a reliable measure of material deprivation developed by Saunders and Chen (2015). Respondents in the original survey were asked to report whether they were deprived of a series of material resources, such as food, shoes, clothes, clean water, pocket money, and a computer. For example, respondents would indicate whether they “do not eat breakfast or lunch to save money”, “do not go out with friends because of not enough pocket money”, and “own[ing] one pair of shoes”. We assigned a score of 1 when any material resource was deprived, and 0 otherwise. The total scores were obtained from 17 dummy items, with a higher score indicating more serious material deprivation.

#### 3.2.2 Family–Adolescent Relationships

The TDCYP provided a 15‐item scale to measure this latent variable, which was adapted from the Family Adaptation and Cohesion Scale (Olson 1986), which showed good internal reliability and construct validity in Taiwanese adolescents (Chiang and Bai 2022; Chiang and Chen 2017). Respondents were asked to report on the quality of their interactions with family members. Example items are: “Family members ask each other for help,” “I can rely on my family when I need help or when I want to hear some honest advice,” and “I can find comfort from my family when I experience setbacks in life.” Each item was scored on a 4‐point Likert scale, ranging from “Very much disagree” (1) to “Very much agree” (4). The total scores ranged from 15 to 60, with higher scores indicating better family–adolescent relationships. Cronbach's *α* of this scale in the present study was 0.94.

#### 3.2.3 Peer Relationships

This latent variable was assessed by a three‐item four‐point Likert scale developed by the TDCYP investigating the quality of adolescents' peer relationships in school life. The specific items are: “My classmates/peers and I share our good and bad moods”, “My classmates/peers and I can understand each other”, “My classmates/peers and I share our daily lives and tell each other our thoughts”. Responses were scored on a scale from “Very much disagree” (1) to “Very much agree” (4). The total scores ranged from 3 to 12, with higher scores indicating better peer relationships. This scale showed good internal reliability in previous studies (Chiang and Chen 2017). Cronbach's *α* of this scale in our study was 0.91.

#### 3.2.4 Teacher–Student Relationships

This latent variable was evaluated by a six‐item four‐point Likert scale developed by the TDCYP and validated in previous studies (Chiang and Chen 2017). Adolescents were asked about the quality of interactions with their teachers. Example items are: “My teacher and I have good interactions,” “I would seek help from my teacher if I were in trouble,” “My teacher treats me better than others in school,” and “My teacher cares about me.” Responses were scored from “Very much disagree” (1) to “Very much agree” (4). The total scores ranged from 6 to 24, with higher scores indicating better teacher–student relationships. Cronbach's *α* of this scale in our study was 0.88.

#### 3.2.5 Psychological Distress

The TDCYP provided a five‐item five‐point Likert scale to measure this latent variable, which was adapted from the Brief Symptom Rating Scale on anxiety and depression symptoms during the last week (depression: feeling depressed or in a low mood; anxiety: feeling tense or high‐strung; interpersonal sensitivity: feeling inferior to others; hostility: feeling easily irritated; and sleep problems: having trouble with falling asleep). This scale is a useful tool for screening mental disorders and is highly valid and reliable in the Taiwanese context (Ahern et al. 2006; Wannasri et al. 2025). Responses were scored from 0 to 4, with higher scores indicating worse psychological distress. This scale was reliable in the present study (Cronbach's *α* = 0.88).

#### 3.2.6 Substance Use

This study used a three‐item scale developed by the TDCYP to evaluate adolescents' substance use. Three dummy items investigated whether or not respondents had recently demonstrated the three prevalent substance use behaviors: smoking cigarettes, drinking alcohol, and chewing betel nuts. In Taiwan, although there is a prohibition on selling betel nut to individuals under 18, it remains the third most popular substance after alcohol and tobacco (Liang et al. 2020). Betel nut chewing is a widespread substance use behavior rooted in the local Taiwanese culture, despite the known health risks, and nearly two million people consume it out of a population of about 23 million (Liang et al. 2020). In the present study, the total scores of the three dummies ranged from 0 to 3, with higher scores indicating more substance use. This scale has been widely used to evaluate adolescent substance use and its associated socioeconomic and family factors in Taiwan (Wu et al. 2015; Yen et al. 2007).

#### 3.2.7 Covariates and Subgroups

Following previous studies using the TDCYP among socioeconomically disadvantaged adolescents, demographic information on sex (0 = female and 1 = male), age, and family characteristics—structure, size, and income—were included as covariates, given that these variables have previously been considered to influence substance use and psychological distress (Saunders and Chen 2015; C. H. Wu et al. 2019). Family structure was coded as 1 and 0, indicating intact and non‐intact families, respectively. Family size and income were extracted from the original responses in the database. Adolescents in the middle or high school systems were included to examine age‐related differences and were coded as 0 and 1, respectively. Sex subgroups were also included in subgroup analyses.

### 3.3 Data Analysis

We computed sample descriptive statistics and correlation coefficients for the included variables. Structural equation modeling (SEM) was then employed to test the mediating effects of the three types of social relationship quality in the mechanisms between adolescents' material deprivation and their outcomes of psychological distress and substance use. The multi‐group SEM was used to explore age and sex differences in the mediation models, using the *lavaan* package in R. Our SEM results were evaluated by the model fit indices below: chi‐square, comparative fit index (CFI), standardized root‐mean square residual (SRMR), and root‐mean‐square of approximation (RMSEA). Researchers have recommended the following cut‐off criteria for acceptable model fit: CFI > 0.90 and SRMR and RMSEA < 0.08 (Hu and Bentler 1998). We further conducted bootstrap analysis using 2000 resamples to estimate the indirect effects. If the 95% bias‐corrected confidence intervals (CI) did not contain zero, the effects were significant (Preacher and Hayes 2008). The subgroup differences in indirect effects were tested by the defined subtraction terms.

## 4 Results

Table 1 shows descriptive statistics in the total sample. Most adolescents (78.03%) were from non‐intact families, and the mean family size was 4.49. Table 2 shows the bivariate correlation coefficients with means and standard deviations.

**TABLE 1:** Descriptive statistics (*N* = 1329).

Variables	Categories	*N*	%
Sex	Female	669	50.34
	Male	660	49.66
Age	Range (10–17)	Mean (SD) = 15.24 (1.38)	
Grade	Middle school	777	58.47
	High school	552	41.53
Family structure	Intact family	292	21.97
	Non‐intact family	1037	78.03
Family size	Range (2–13)	Mean (SD) = 4.49 (1.50)	
Family income (NT$)	No income	139	10.46
	1–5000	64	4.82
	5000–10,000	150	11.29
	10,000–15,000	209	15.73
	15,000–20,000	252	18.96
	20,000–25,000	268	20.17
	25,000–30,000	120	9.03
	30,000–35,000	75	5.64
	35,000–40,000	23	1.73
	40,000–45,000	13	0.98
	45,000 and above	16	1.20

**TABLE 2:** Bivariate correlations.

	M	SD	1	2	3	4	5	6
1. MD	2.02	1.61	1.00					
2. FR	43.62	8.08	−0.10***	1.00				
3. PR	9.26	1.77	−0.16***	0.30***	1.00			
4. TR	17.01	3.05	−0.05*	0.32***	0.34***	1.00		
5. PD	9.25	4.29	0.21***	−0.29***	−0.10***	−0.13***	1.00	
6. SU	0.22	0.57	−0.03	−0.12***	−0.01	−0.08***	0.07***	1.00

*Note:* *p < 0.05, ***p < 0.001.

Abbreviations: FR: Family–Adolescent Relationships, M: Mean, MD: Material Deprivation, PD: Psychological Distress, PR: Peer Relationships, SD: Standard deviation, SU: Substance Use, TR: Teacher–Student Relationships.

### 4.1 Mediating Effects in the Total Sample

**FIGURE 1:**
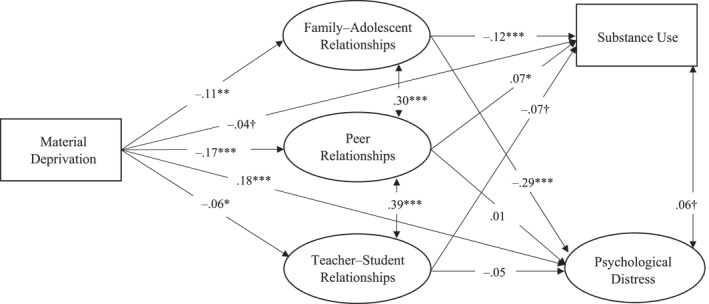
Pathway coefficients of the structural equation model in the total sample. Coefficients are standardized. Model fit indices: CFI = 0.942, GFI = 0.905, RMSEA = 0.043, SRMR = 0.029. Bootstrapping: 2000 times. Covariates: sex, age, family structure, family size, family income. For clarity of presentation, the correlation between family–adolescent relationships and teacher–student relationships is not shown in the figure, which is 0.34***. †*p* < 0.1, *p < 0.05, **p < 0.01, ***p < 0.001.

Figure 1 and Table 3 present our SEM results in the total sample. Our model fits the data well: CFI = 0.942, GFI = 0.905, RMSEA = 0.043, SRMR = 0.029. After controlling for demographics and family characteristics, material deprivation was negatively associated with the quality of family–adolescent relationships, teacher–student relationships, peer relationships (*β* = −0.11, *p* < 0.01; *β* = −0.06, *p* < 0.05; and *β* = −0.17, *p* < 0.001, respectively), and substance use (*β* = −0.04, *p* > 0.05), while positively associated with psychological distress (*β* = 0.18, *p* < 0.001). The quality of the three types of social relationships had different magnitudes and directions in their associations with psychological distress and substance use. The quality of family–adolescent relationships was significantly and negatively associated with psychological distress (*β* = −0.29, *p* < 0.001) and substance use (*β* = −0.12, *p* < 0.001). The quality of teacher–student relationships was also negatively associated with psychological distress (*β* = −0.05, *p* > 0.05) and substance use (*β* = −0.07, *p* > 0.05), while the magnitudes were weak and nonsignificant. The quality of peer relationships had a significant positive association with substance use (*β* = 0.07, *p* < 0.05), yet a nonsignificant association with psychological distress (*β* = 0.01, *p* > 0.05). Sex and age were significantly associated with psychological distress and substance use. Family characteristics in structure, size, and income had nonsignificant associations with psychological distress, while family size and income had positive associations with substance use at the level of *p* < 0.05 (*β* = 0.07 and 0.06).

**TABLE 3:** Pathway coefficients of the structural equation model in the total sample.

Variables	Model 1: Family–Adolescent relationships			Model 2: Peer relationships			Model 3: Teacher–Student relationships			Model 4: Psychological distress			Model 5: Substance use		
	*β*	SE	*p*	*β*	SE	*p*	*β*	SE	*p*	*β*	SE	*p*	*β*	SE	*p*
Sex	0.044	0.024	0.122	0.125	0.033	0.000	0.018	0.027	0.538	0.134	0.041	0.000	−0.136	0.032	0.000
Age	−0.042	0.009	0.130	0.003	0.012	0.917	−0.011	0.009	0.703	0.106	0.015	0.000	0.065	0.011	0.013
Family structure	0.036	0.028	0.196	0.031	0.041	0.313	0.035	0.033	0.238	−0.003	0.051	0.910	−0.033	0.035	0.188
Family size	0.007	0.009	0.819	−0.009	0.012	0.779	0.013	0.009	0.653	−0.004	0.014	0.897	0.069	0.012	0.021
Family income	−0.005	0.006	0.851	0.005	0.007	0.870	−0.022	0.006	0.435	0.014	0.009	0.610	0.064	0.007	0.022
Material deprivation	−0.109	0.009	0.001	−0.165	0.010	0.000	−0.064	0.009	0.043	0.176	0.013	0.000	−0.042	0.008	0.074
Family–adolescent relationships										−0.294	0.071	0.000	−0.123	0.047	0.000
Peer relationships										0.013	0.051	0.743	0.067	0.032	0.030
Teacher–student relationships										−0.050	0.066	0.225	−0.070	0.046	0.055

Abbreviations: *β*: standardized coefficients; SE: standard error; *p*: *p*‐values of significance tests.

Table 4 shows bootstrapping results regarding the mediating effects of the social relationship quality. The indirect effects of material deprivation on substance use via the quality of family–adolescent relationships, teacher–student relationships, and peer relationships were 0.013 (95% CI = [0.002, 0.009], SE = 0.002), 0.005 (95% CI = [0.000, 0.004], SE = 0.001), and −0.011 (95% CI = [–0.008, −0.000], SE = 0.002). Meanwhile, the indirect effects of material deprivation on psychological distress via the quality of family–adolescent relationships, teacher–student relationships, and peer relationships were 0.032 (95% CI = [0.006, 0.025], SE = 0.005), 0.003 (95% CI = [–0.001, 0.005], SE = 0.002), and −0.002 (95% CI = [–0.007, 0.005], SE = 0.003).

**TABLE 4:** Bootstrapping mediating effects of social relationships in the total sample.

Model paths	Effect	BootSE	BootLLCI	BootULCI
MD –> FR –> PD	0.032	0.005	0.006	0.025
MD –> PR –> PD	−0.002	0.003	−0.007	0.005
MD –> TR –> PD	0.003	0.002	−0.001	0.005
MD –> FR –> SU	0.013	0.002	0.002	0.009
MD –> PR –> SU	−0.011	0.002	−0.008	−0.000
MD –> TR –> SU	0.005	0.001	0.000	0.004

*Note:* Effect: standardized indirect effects; BootSE, BootLLCI, & BootULCI: standard error, 95% bias‐corrected lower/upper limit confidence interval, generated from 2000 times bootstrapping analysis.

Abbreviations: FR: Family–Adolescent Relationships, MD: Material Deprivation, PD: Psychological Distress, PR: Peer Relationships, SU: Substance Use, TR: Teacher–Student Relationships.

### 4.2 Age and Sex Differences

**FIGURE 2:**
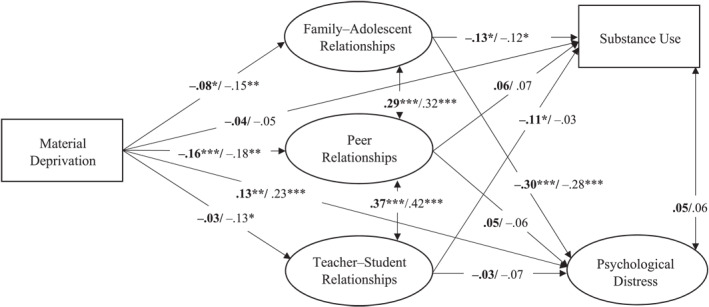
Pathway coefficients of the structural equation model in the grade sub‐samples. Standardized coefficients of middle school adolescents (*N* = 777) are presented in bold, followed by those of high school adolescents (*N* = 552). Model fit indices: CFI = 0.934, GFI = 0.980, RMSEA = 0.046, SRMR = 0.034. Bootstrapping: 2000 times. Covariates: sex, age, family structure, family size, family income. For clarity of presentation, the correlations between family–adolescent relationships and teacher–student relationships among middle and high school adolescents are not shown in the figure, which are 0.37*** and 0.31***, respectively. *p < 0.05, **p < 0.01, ***p < 0.001.

The multi‐group SEM results in the age sub‐samples were presented in Figure 2 and Supporting Information S1: Appendix Table 1. The multi‐group model fits the data well as all indices satisfy the corresponding criteria. Material deprivation had slightly stronger associations with the three types of social relationships and psychological distress among high school adolescents than middle school adolescents. Meanwhile, the quality of social relationships had slightly stronger associations with substance use among middle‐school adolescents than their counterparts in high school. The quality of teacher–student relationships remained negatively associated with the two adolescent outcomes, and the marginal magnitude of the association with substance use was stronger among middle school adolescents (*β* = −0.11, *p* < 0.05).

**FIGURE 3:**
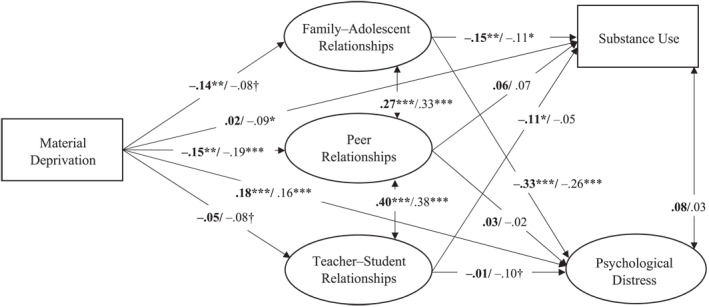
Pathway coefficients of the structural equation model in the sex sub‐samples. Standardized coefficients of female adolescents (*N* = 669) are presented in bold, followed by those of male adolescents (*N* = 660). Model fit indices: CFI = 0.939, GFI = 0.981, RMSEA = 0.045, SRMR = 0.035. Bootstrapping: 2000 times. Covariates: sex, age, family structure, family size, family income. For clarity of presentation, the correlations between family–adolescent relationships and teacher–student relationships among middle and high school adolescents are not shown in the figure, which are 0.34*** and 0.35***, respectively. †*p* < 0.1, *p < 0.05, **p < 0.01, ***p < 0.001.

Figure 3 and Supporting Information S1: Appendix Table 2 show the multi‐group SEM results in our gender sub‐samples. The associations of the quality of family–adolescent relationships with material deprivation, substance use, and psychological distress were stronger among female adolescents. The quality of teacher–student relationships had a stronger association with substance use among female adolescents (*β* = −0.11, *p* < 0.05), while a stronger association with psychological distress among male adolescents (*β* = −0.10, *p* < 0.1).

Supporting Information S1: Appendix Table 3 shows the bootstrapping results regarding the mediating effects of the quality of social relationships generated from 2000 resamples in age and sex subgroups. The mediating effects of the quality of family–adolescent relationships were significant across middle and high school students and female and male adolescents. The mediating effects of the quality of peer relationships and teacher–student relationships were nonsignificant among the four subgroups. Notably, group differences in the indirect effects between age sub‐samples and sex sub‐samples were nonsignificant.

## 5 Discussion

This study used data from 1329 Taiwanese adolescents living in poverty and receiving social assistance to test the mediating roles of the social relationship quality in the associations between material deprivation, psychological distress, and substance use. Multi‐group SEM was utilized to further examine age and sex differences in the mediating models. Empirical findings were discussed for elucidating interrelationships between material deprivation, psychological distress, and substance use among socioeconomically disadvantaged adolescents, thus informing adolescent mental and behavioral health practices.

### 5.1 The Overall Mediation Model

Our results showed that material deprivation was associated with social relationships, substance use, and psychological distress. Its significant negative associations with the quality of three types of social relationships and positive associations with psychological distress are consistent with previous theories and studies showing that adolescents deprived of material and financial resources in family contexts or from lower socioeconomic status may demonstrate lower levels of social competencies and higher levels of emotional sufferings (Conger et al. 2010; Gross‐Manos and Ben‐Arieh 2017). This finding further supports socioeconomic inequalities in adolescent health, particularly among populations living in poverty and receiving social assistance (Adler and Stewart 2010; J. K. Chen, Wang, et al. 2021; Liu and Chen 2024; Makarios et al. 2017).

The study found that the quality of the three types of social relationships significantly mediated the associations between material deprivation and substance use. This finding provides empirical evidence supporting previous theoretical discussions, which have consistently argued that the quality of social relationships plays a mediating role in the associations between socioeconomic indicators and adolescent outcomes (Adler and Stewart 2010; Yoshikawa et al. 2012). Specifically, our study echoes previous research and suggests that good‐quality family–adolescent relationships and teacher–student relationships link with lower levels of adolescent substance use (Buttram et al. 2012). In addition, the positive association between peer relationship quality and substance use aligns with previous studies showing the promotive role of peer influences or affiliation with delinquent peers in increasing adolescent substance use and misconduct at school (J. K. Chen, Yang, et al. 2023; Coley et al. 2017; Han and Chen 2025). Taken together, our findings above are reasonable and support the mediating effects of the social relationship quality in the interrelationships between material deprivation and adolescent outcomes. The positive and negative associations of the quality of social relationships with substance use further imply that the roles of adolescents' interactions may be different across sources, such as families, teachers, and peers.

The results also showed that the quality of family–adolescent relationships instead of peer and teacher–student relationships significantly mediated the association of material deprivation with psychological distress. This mediating role is distinctive from that of the models with substance use as the outcome, while it is consistent with previous literature documenting the cumulative stress processes from material deprivation to adolescents' poor social relationships and emotional problems (Gross‐Manos and Ben‐Arieh 2017). Indeed, the consistently significant mediating effects of the quality of family–adolescent relationships echo our empirical results, showing that the relationships had stronger associations with the adolescent outcomes, compared with the quality of peer and teacher–student relationships. These findings support the perspectives of family theorists rather than developmental or educational psychologists, highlighting that the quality of family–adolescent relationships could be a more salient pathway to adolescent health outcomes (J. K. Chen et al. 2023; Conger et al. 2010; He et al. 2025; Steinberg 2001).

### 5.2 Subgroup Differences

We found that the mediating effects of the family–adolescent relationship quality remained significant across all age and sex subgroups analyses. Moreover, group differences in indirect effects between age and between sex sub‐samples were all nonsignificant. This pattern is consistent with previous studies on the mediating roles of social relationships concerning the impact of material deprivation on children's outcomes (J. K. Chen et al. 2023). Generally, our findings support that the interrelationships between material deprivation and adolescent substance use and psychological distress could be applicable in both sex and age sub‐samples, particularly regarding the mediating roles of the quality of family–adolescent relationships.

It is worth noting that certain pathways in the model had slightly different magnitudes across sex and age subgroups, although the differences were not statistically significant. For example, the associations of the quality of family–adolescent relationships with material deprivation, substance use, and psychological distress were stronger among female adolescents. This finding suggests that harmonious family relationships may be important for female adolescents in diminishing their risks of substance use and psychological distress, as informed by similar studies (J. K. Chen et al. 2023), while this speculation for practical relevance needs more robust examinations by longitudinal studies.

Second, the associations of material deprivation with the social relationship quality and psychological distress were stronger among high school students, while the associations of the quality of social relationships with substance use were stronger among middle school students. Although the differences were not statistically significant, it may suggest that the roles of social relationships could have developmental heterogeneity that should be applied well in efforts to reduce substance use for middle school students and psychological distress for high school students. Indeed, previous research has provided evidence supporting our differential effects of social relationships, that is, a higher prevalence of substance use in middle school and an upward trend of emotional distress varied by ages (Dong et al. 2024; O’Malley et al. 2006; Tian et al. 2021). Following this, more examinations are needed to further support and extend our preliminary findings. Overall, the nuanced differences in our subgroup results highlight that although the quality of social relationships may generally link with adolescent health and well‐being, age‐ and gender‐specific insights should be considered for empirical explorations and practical applications.

### 5.3 Theoretical Contributions

This study makes multifaceted research contributions. First, it supports the idea of material deprivation as a socioeconomic determinant of adolescent mental and behavioral health by supporting its significant direct associations with substance use and psychological distress and indirect effects via the quality of social relationships. This contribution supports previous theoretical perspectives on socioeconomic determinants of health (Adler and Stewart 2010; Braveman et al. 2011). Conventional socioeconomic determinants include several indicators, such as income, education, and employment, while these have been criticized not to reflect the actual situations of socioeconomic position in the children and adolescent world (J. K. Chen, Wang, et al. 2021; Gross‐Manos and Ben‐Arieh 2017). The adolescent‐perceived scarcity and lack of material resources reflect both socioeconomic conditions and their needs, which have been increasingly used in the recent literature linking with health outcomes (Khin et al. 2024). Following this, our study further enriches empirical evidence on its links with psychological distress and substance use.

Second, this study enriches the existing arguments on the psychosocial pathways in the socioeconomic determinants of adolescent health by exploring the mediating roles of the quality of social relationships within family and school contexts. Specifically, our findings indicate that good‐quality family–adolescent relationships and teacher–student relationships may serve as a resilience factor in explaining the interrelationships between material deprivation and adolescent psychological distress and substance use. Before the proposal of psychosocial pathways, such as social relationships, early theoretical discussions about socioeconomic determinants of health had focused on structural factors relevant to social gradient and economic inequality (Adler and Stewart 2010; Braveman et al. 2011). Our study supports the importance of psychosocial pathways in explaining the direct links between socioeconomic determinants and health outcomes, while further highlighting that adolescents' social interactions with family members, teachers, and peers are crucial contexts for psychosocial functioning at this transitional stage across the life course. The more salient role of the quality of family–adolescent relationships in this study may also contribute to the literature by emphasizing family dynamics and interactions in psychosocial pathways linking socioeconomic determinants with adolescent mental and behavioral health.

Third, this study extends the mediating roles of the quality of social relationships to a behavioral outcome, that is, substance use, and a vulnerable minority group, that is, socioeconomically disadvantaged adolescents. This contribution enriches the literature of mediating mechanisms between material deprivation, social relationships, and adolescent outcomes, which has previously focused on psychological outcomes and mainstream or socially privileged adolescent populations (J. K. Chen et al. 2023; Lau and Bradshaw 2016). Generally, our results suggest that the mediation model of social relationship quality can be similarly applied to substance use and socioeconomically disadvantaged adolescents. Our results further inform future discussions and examinations about the nuanced different effects on behavioral problems from psychological outcomes and the developmental heterogeneity in the mediating mechanisms across age and sex subgroups.

### 5.4 Practical Implications

This study provides practical implications for both policymakers and service practitioners. Some policies, regulations, and interventions for low‐income adolescents and their families, such as cash transfers, housing allowances, and social assistance, merely targeting material resources for poverty alleviation may not be effective and even induce welfare stigma and reliance, as research evidence shows (K. M. Chen et al. 2014; Courtin et al. 2019; Viitasalo et al. 2023). An early program introduced by the TFCF in Taiwan is an example, with adolescents receiving welfare resources showing lower levels of educational aspirations and performances (Saunders and Chen 2015). Our findings support the idea that family dynamics may be a suitable target for interventions to counteract cumulative socioeconomic disadvantages faced by adolescents. Some innovative policies and regulations have integrated social interventions targeting family dynamics, adolescent development, and inter‐generational bonding beyond separate cash transfers (K. M. Chen et al. 2014; J. K. Chen et al. 2021). As such, practitioners may leverage the good‐quality family relationships and socioeconomic interventions to comprehensively address adolescent mental and behavioral health issues.

Our findings also inform relationship‐targeted programs in the family and school contexts. Given the important role of social interactions with family members, teachers, and peers across age and sex subgroups, practitioners may cultivate good‐quality social interactions in promotion, prevention, and intervention programs for adolescents faced by material deprivation and other socioeconomic disadvantages, thus addressing their possible mental and behavioral health issues. For example, as advocated and practiced by the TFCF for years, conventional socioeconomic support (such as social assistance to adolescents in poverty) and family‐based psychosocial support (such as parent‐child parallel group work, parenting education, psychological counseling, and social network empowerment) should be combined in policy design and service delivery.

Moreover, our study identifies complex mechanisms relevant to various sources of social relationships and adolescent outcomes, which covers multiple subjects, disciplines, contexts, and systems. Thus, policy and service programs should also be designed by multidisciplinary researchers and practitioners, such as social workers, educators, family therapists, and juvenile justice professionals. Indeed, the database used in this study, the TDCYP by the Taiwan Fund for Children and Families (2014), is also co‐produced by researchers and practitioners dedicated to poverty alleviation and family well‐being.

In addition, our subgroup analyses inform appropriate population‐specific strategies. The nonsignificant group differences in the mediating effects suggest that relationship‐targeted programs may be delivered to female and male adolescents in middle schools. Some nuanced differences warranting further empirical and practical explorations also suggest possible directions that high‐quality family–adolescent and teacher–student relationships may be useful in addressing substance use for middle school or female adolescents and reducing psychological distress for high school or male adolescents.

### 5.5 Limitations and Future Research Directions

This study still has limitations, providing directions for future studies to improve. First, we should exercise caution when interpreting our findings because the results were drawn from cross‐sectional data. Given the lack of longitudinal data for our variables at this stage, the findings are correlational and can only indicate potential interrelationships. Although our conceptual model was informed by theoretical perspectives and previous empirical research, the temporal pathways linking material deprivation, social relationships, and adolescent outcomes should be rigorously examined using longitudinal designs. For instance, psychological distress and substance use may have distinct temporal dynamics as well as associations, even though they were treated here as concurrent and related indicators of internalizing and externalizing problems. Generally, although this correlational study contributes to understanding the roles of social relationships in explaining the associations between material deprivation and specific substance use among socioeconomically disadvantaged adolescents, we recommend that future research can replicate and extend our preliminary results with more intensive longitudinal data. Second, variables in this study are derived from self‐report responses—an approach that may induce estimating bias, given that some adolescents may overstate their deprivation conditions and underreport their substance use behaviors (J. K. Chen et al. 2023). Future researchers are thus advised to use multisource data for triangulation, such as objective records or reports from parents and teachers. Third, some coefficients in our study were small and marginal, which may limit the practical significance of relevant findings. Indeed, some studies among Chinese samples also found small magnitudes in the associations between family‐related socioeconomic resources and adolescent development (J. K. Chen, Wang, et al. 2021; Liu and Chen 2024). In addition, given that the effects of socioeconomic indicators on health outcomes may cumulate over time, determining the true associations with substance use and psychological distress warrants investigations on later developmental periods (Lee et al. 2017). Thus, future research is also advised to examine our proposed mechanisms in the samples of late adolescence or emerging adulthood.

## Author Contributions

**Zewei Liu:** conceptualization, methodology, data curation, formal analysis, writing – original draft, software, visualization. **Ji‐Kang Chen:** supervision, conceptualization, methodology, validation, writing – review and editing, resources, project administration, funding acquisition.

## Funding

International Research Collaboration Fund granted by the Department of Social Work, The Chinese University of Hong Kong (Grant 19231105).

## Ethics Statement

This study was a secondary data analysis. The data we received and analyzed for this study were completely anonymized and did not contain any personally identifiable information. All procedures performed in the study involving human participants were described as being in accordance with the ethical standards of the institutional and/or national research committee and with the 1964 Helsinki Declaration and its later amendments or comparable ethical standards.

## Conflicts of Interest

The authors declare no conflicts of interest.

## Supporting information


Supporting Information S1

## Data Availability

Further details on these data, please see Taiwan Fund for Children and Families (2014). Taiwan Database of Children and Youth in Poverty: The Panel Study in 2013 (D00116) [data file]. Available from Survey Research Data Archive, Academia Sinica. https://doi.org/10.6141/TW‐SRDA‐D00116‐1.
