# Supplementary material for: Material Deprivation, Psychological Distress, and Substance Use Among Socioeconomically Disadvantaged Adolescents: The Mediating Roles of Social Relationships
Source: Behav Sci Law. 2026 Mar 24;44(4):519–30. doi: 10.1002/bsl.70058 (PMC13436466; doi:10.1002/bsl.70058)
Supplement: Supplementary file 1 [file BSL-44-519-s001.docx]

| **Appendix Table 1** Pathway coefficients of the structural equation model in the grade sub-samples | | | | | | | | | | | | | | | |
| --- | --- | --- | --- | --- | --- | --- | --- | --- | --- | --- | --- | --- | --- | --- | --- |
| Variables | Model 1: Family–Adolescent Relationships | | | Model 2: Peer Relationships | | | Model 3: Teacher–Student Relationships | | | Model 4: Psychological Distress | | | Model 5: Substance Use | | |
|  | β | SE | p | β | SE | p | β | SE | p | β | SE | p | β | SE | p |
| *Middle school adolescents (N = 777)* | | | | | | | | | | | | | | | |
| Sex | .097 | .031 | .009 | .167 | .044 | .000 | .035 | .038 | .356 | .157 | .053 | .000 | –.062 | .035 | .071 |
| Age | –.019 | .015 | .608 | .050 | .020 | .167 | .026 | .019 | .503 | .069 | .026 | .062 | .002 | .017 | .952 |
| Family structure | .035 | .035 | .324 | .027 | .057 | .495 | .043 | .043 | .231 | –.017 | .064 | .655 | –.020 | .038 | .514 |
| Family size | –.020 | .011 | .612 | –.001 | .016 | .980 | .009 | .012 | .804 | –.021 | .018 | .573 | .013 | .013 | .739 |
| Family income | .052 | .007 | .175 | .016 | .010 | .669 | –.020 | .009 | .602 | .013 | .013 | .727 | .043 | .009 | .235 |
| MD | –.082 | .010 | .022 | –.155 | .013 | .000 | –.025 | .012 | .496 | .126 | .017 | .001 | –.044 | .009 | .121 |
| FR |  |  |  |  |  |  |  |  |  | –.301 | .090 | .000 | –.133 | .063 | .010 |
| PR |  |  |  |  |  |  |  |  |  | .049 | .065 | .362 | .062 | .037 | .145 |
| TR |  |  |  |  |  |  |  |  |  | –.028 | .086 | .633 | –.106 | .053 | .041 |
| *High school adolescents (N = 552)* | | | | | | | | | | | | | | | |
| Sex | –.023 | .039 | .605 | .057 | .045 | .202 | –.015 | .037 | .741 | .112 | .065 | .009 | –.216 | .056 | .000 |
| Age | .008 | .030 | .857 | .006 | .031 | .883 | –.052 | .028 | .257 | .041 | .047 | .313 | –.062 | .051 | .236 |
| Family structure | .035 | .050 | .452 | .034 | .054 | .440 | .016 | .051 | .753 | .015 | .085 | .749 | –.051 | .067 | .235 |
| Family size | .053 | .015 | .278 | –.018 | .017 | .722 | .016 | .013 | .745 | .013 | .025 | .792 | .116 | .021 | .014 |
| Family income | –.080 | .009 | .086 | –.009 | .009 | .826 | –.024 | .008 | .585 | .012 | .014 | .767 | .090 | .012 | .033 |
| MD | –.148 | .014 | .005 | –.184 | .016 | .001 | –.127 | .013 | .020 | .229 | .020 | .000 | –.045 | .016 | .276 |
| FR |  |  |  |  |  |  |  |  |  | –.278 | .093 | .000 | –.121 | .071 | .013 |
| PR |  |  |  |  |  |  |  |  |  | –.060 | .082 | .271 | .069 | .061 | .141 |
| TR |  |  |  |  |  |  |  |  |  | –.072 | .102 | .190 | –.031 | .087 | .578 |
| ***Notes.*** β: standardized coefficients; SE: standard error; p: p values of significance tests. MD: Material Deprivation, FR: Family–Adolescent Relationships, PR: Peer Relationships, TR: Teacher–Student Relationships. | | | | | | | | | | | | | | | |

| **Appendix Table 2** Pathway coefficients of the structural equation model in the sex sub-samples | | | | | | | | | | | | | | | |
| --- | --- | --- | --- | --- | --- | --- | --- | --- | --- | --- | --- | --- | --- | --- | --- |
| Variables | Model 1: Family– Adolescent Relationships | | | Model 2: Peer Relationships | | | Model 3: Teacher–Student Relationships | | | Model 4: Psychological Distress | | | Model 5: Substance Use | | |
|  | β | SE | p | β | SE | p | β | SE | p | β | SE | p | β | SE | p |
| *Female adolescents (N = 669)* | | | | | | | | | | | | | | | |
| Age | –.091 | .013 | .026 | –.076 | .016 | .060 | –.034 | .014 | .426 | .081 | .021 | .038 | –.002 | .012 | .955 |
| Family structure | .002 | .040 | .950 | –.016 | .053 | .706 | .028 | .045 | .511 | –.012 | .073 | .770 | –.080 | .034 | .013 |
| Family size | .043 | .013 | .352 | .024 | .015 | .553 | .027 | .014 | .571 | –.009 | .021 | .823 | .046 | .012 | .258 |
| Family income | .005 | .008 | .897 | .020 | .009 | .591 | –.018 | .008 | .641 | –.004 | .013 | .919 | .023 | .009 | .596 |
| MD | –.137 | .012 | .002 | –.152 | .015 | .001 | –.051 | .012 | .244 | .184 | .020 | .000 | .015 | .011 | .683 |
| FR |  |  |  |  |  |  |  |  |  | –.325 | .098 | .000 | –.152 | .057 | .005 |
| PR |  |  |  |  |  |  |  |  |  | .030 | .074 | .563 | .064 | .041 | .203 |
| TR |  |  |  |  |  |  |  |  |  | –.004 | .097 | .943 | –.108 | .055 | .050 |
| *Male adolescents (N = 660)* | | | | | | | | | | | | | | | |
| Age | .005 | .012 | .892 | .080 | .018 | .068 | .012 | .013 | .762 | .143 | .018 | .000 | .115 | .018 | .002 |
| Family structure | .073 | .042 | .073 | .072 | .060 | .100 | .041 | .049 | .324 | .005 | .070 | .898 | –.008 | .067 | .840 |
| Family size | –.027 | .011 | .507 | –.038 | .017 | .411 | –.002 | .012 | .965 | .002 | .019 | .960 | .085 | .020 | .061 |
| Family income | –.020 | .008 | .648 | –.012 | .010 | .767 | –.023 | .009 | .585 | .033 | .013 | .430 | .093 | .012 | .013 |
| MD | –.084 | .012 | .066 | –.185 | .014 | .000 | –.078 | .014 | .090 | .162 | .018 | .000 | –.085 | .014 | .010 |
| FR |  |  |  |  |  |  |  |  |  | –.256 | .089 | .000 | –.109 | .076 | .020 |
| PR |  |  |  |  |  |  |  |  |  | –.022 | .071 | .696 | .060 | .048 | .127 |
| TR |  |  |  |  |  |  |  |  |  | –.096 | .084 | .094 | –.048 | .071 | .341 |
| ***Notes.*** β: standardized coefficients; SE: standard error; p: p values of significance tests. MD: Material Deprivation, FR: Family–Adolescent Relationships, PR: Peer Relationships, TR: Teacher–Student Relationships. | | | | | | | | | | | | | | | |

| **Appendix Table 3** Bootstrapping mediating effects in grade and sex sub-samples | | | | |
| --- | --- | --- | --- | --- |
| Model paths | Effect | BootSE | BootLLCI | BootULCI |
| *Middle school adolescents (N = 777)* | | | | |
| MD –> FR –> PD | .025 | .005 | .002 | .022 |
| MD –> PR –> PD | –.008 | .004 | –.012 | .004 |
| MD –> TR –> PD | .001 | .001 | –.002 | .003 |
| MD –> FR –> SU | .011 | .002 | .000 | .009 |
| MD –> PR –> SU | –.010 | .002 | –.008 | .001 |
| MD –> TR –> SU | .003 | .001 | –.002 | .004 |
| *High school adolescents (N = 552)* | | | | |
| MD –> FR –> PD | .041 | .008 | .005 | .036 |
| MD –> PR –> PD | .011 | .005 | –.004 | .017 |
| MD –> TR –> PD | .009 | .004 | –.002 | .013 |
| MD –> FR –> SU | .018 | .004 | .001 | .016 |
| MD –> PR –> SU | –.013 | .004 | –.013 | .001 |
| MD –> TR –> SU | .004 | .003 | –.004 | .008 |
| *Differences in indirect effects between middle and high school adolescents (middle* – *high)* | | | | |
| MD –> FR –> PD | –.016 | .009 | –.026 | .010 |
| MD –> PR –> PD | –.019 | .006 | –.023 | .003 |
| MD –> TR –> PD | –.008 | .004 | –.014 | .003 |
| MD –> FR –> SU | –.007 | .004 | –.013 | .004 |
| MD –> PR –> SU | .003 | .004 | –.006 | .011 |
| MD –> TR –> SU | –.001 | .003 | –.008 | .006 |
| *Female adolescents (N = 669)* | | | | |
| MD –> FR –> PD | .045 | .008 | .007 | .038 |
| MD –> PR –> PD | –.005 | .004 | –.010 | .006 |
| MD –> TR –> PD | .000 | .002 | –.004 | .004 |
| MD –> FR –> SU | .021 | .003 | .001 | .013 |
| MD –> PR –> SU | –.010 | .002 | –.008 | .001 |
| MD –> TR –> SU | .005 | .002 | –.001 | .005 |
| *Male adolescents (N = 660)* | | | | |
| MD –> FR –> PD | .022 | .005 | .000 | .021 |
| MD –> PR –> PD | .004 | .005 | –.008 | .012 |
| MD –> TR –> PD | .008 | .003 | –.001 | .011 |
| MD –> FR –> SU | .009 | .003 | .000 | .011 |
| MD –> PR –> SU | –.011 | .003 | –.012 | .001 |
| MD –> TR –> SU | .004 | .002 | –.002 | .006 |
| *Differences in indirect effects between female and male adolescents (female* – *male)* | | | | |
| MD –> FR –> PD | .023 | .009 | –.006 | .032 |
| MD –> PR –> PD | –.009 | .006 | –.016 | .008 |
| MD –> TR –> PD | –.007 | .004 | –.012 | .003 |
| MD –> FR –> SU | .012 | .004 | –.006 | .010 |
| MD –> PR –> SU | .002 | .004 | –.006 | .010 |
| MD –> TR –> SU | .002 | .003 | –.005 | .005 |
| ***Notes.*** MD: Material Deprivation, FR: Family–Adolescent Relationships, PR: Peer Relationships, TR: Teacher–Student Relationships, PD: Psychological Distress, SU: Substance Use. Effect: standardized indirect effects; BootSE, BootLLCI, & BootULCI: standard error, 95% bias–corrected lower/upper limit confidence interval, generated from 2000 times bootstrapping analysis. | | | | |
